# Selection of superior bifidobacteria in the presence of rotavirus

**DOI:** 10.1590/1414-431X20165562

**Published:** 2016-11-10

**Authors:** G. Alp Avci

**Affiliations:** Department of Molecular Biology and Genetics, Molecular Microbiology and Biotechnology, Faculty of Science and Arts, Hitit University, Corum, Turkey

**Keywords:** Rotavirus, Bifidobacteria, EPS production, Cholesterol elimination, Bile salt deconjugation

## Abstract

The main purpose of this study was to investigate bifidobacteria flora in fecal samples from children with rotavirus infection and determine the significance of their selected probiotic properties for improvement of health status. Enzyme-linked immunosorbent assay was used to identify rotavirus antigen in fecal samples from 94 patients with gastroenteritis and from 30 without gastroenteritis. Bifidobacteria were identified by selective media, gram reaction, colony morphology, fructose-6-phosphate phosphoketolase enzyme activity and classical identification tests. Exopolysaccharide (EPS) production was identified by phenol-sulphuric acid method. The modified method was then used to identify the quantity of taurocholic and glycocholic acid deconjugation and cholesterol elimination of the strains. Thirty-five of the 94 fecal samples were found positive for rotavirus antigen (37.23%). Bifidobacteria were identified in 59 of the samples. The EPS production ranges were 29.56-102.21 mg/L. The cholesterol elimination rates ranged between 8.36-39.22%. Furthermore, a positive and strong correlation was determined between EPS production and the presence of cholesterol (*r*=0.984, P<0.001). The deconjugation rates for the sodium glycocholate group was higher than the sodium taurocholate group. Rotavirus (+) bifidobacteria strains had higher EPS production, deconjugation rate and cholesterol elimination compared to bifidobacteria strains isolated from children in the rotavirus (-) sample and without gastroenteritis. Significant differences were observed among groups in all parameters (P<0.05). Given the increased number of rotavirus cases in Turkey and worldwide, it is very important to add superior bifidobacteria in the diets of infected children to improve the intestinal and vital functions.

## Introduction

Rotavirus is the leading cause of acute gastroenteritis among children and neonates, and accounts for an estimated 2 million hospitalizations per year worldwide (1,2). The infection results in a profuse watery diarrhea lasting 2 to 7 days with loss of fluid and electrolytes (3). Secretory immunoglobulin A and probiotics in milk during the lactation period are very important for the protection against enteric infection factors including rotavirus (4,5). Worldwide studies reported on the importance of probiotic microorganisms especially for children under 5 years of age. The most important benefits of probiotic microorganisms include the prevention of several infections, allergic disorders, diarrhea, and inflammatory diseases (6,7). Above all, bifidobacteria play an essential role in the prevention of pathogen microorganisms infection and in the regulation of the intestinal flora due to its probiotic properties. The presence of bifidobacteria in the intestines is a sign of a healthy microbiota (5).

In recent years, several microbiome studies showed the importance of probiotic microorganisms. Therefore, their metabolic functions in terms of the benefits for human health should be studied. Especially, exopolysaccharide (EPS) production by probiotic microorganisms increases and localizes the intestinal adhesion of these microorganisms (8). Besides, EPS production increases gastric acidity and bile tolerance of the microorganisms, and plays an essential role in the protection against pathogenic microorganisms infection (9).

Bile salt deconjugation by intestinal microbiota is very important to decrease the levels of serum cholesterol (10). The deconjugation process is performed by bile salt hydrolase (BSH) enzyme produced by several microorganisms including bifidobacteria and lactobacilli (11,12). It is hypothesized that the deconjugation of bile salts may lead to a reduction in serum cholesterol by reducing the absorption of cholesterol through the intestinal lumen, decreasing the enterohepatic circulation of bile acids, increasing the production of hepatic bile acids, and inducing the precipitation of cholesterol with free bile acids in the intestinal acidic medium (10). Thereby, bile salt deconjugation reduces cholesterol solubility and absorption of cholesterol through the intestinal lumen.

In the light of this literature review, our study aimed to compare bifidobacteria isolated from children with or without rotavirus by identifying probiotic abilities including EPS production, cholesterol elimination and bile salt deconjugation.

## Material and Methods

### Ethics

This study was approved by the Ethics Committee of Ondokuz Mayıs University (Protocol #87/2012).

### Determination of rotavirus positivity

A total of 94 fecal samples from children under 5 years of age with complaints of vomiting, diarrhea, abdominal pain and fever were included in the study between August 2013 and September 2014. Rotavirus group A antigen (Premier Rotaclone, Enzyme-Immunoassay kit, Meridian Diagnostics, Inc., USA) was used with ELISA method to identify rotavirus antigen in fecal samples.

### Isolation, culture conditions and identification of bifidobacteria

The Hadadji et al. (13) method modified by Alp and Aslim (5) was performed for the isolation of bifidobacteria in rotavirus positive (GRV+) and negative (GRV-) fecal samples from children with gastroenteritis and in fecal samples from healthy children without gastroenteritis (WG). One gram of each fecal sample was diluted with 9 mL NaCl (0.9%) in 0.2% L-cysteine-HCl (Merck, Germany) and vortexed for 2 min. Following serial dilutions, 100 µL of bifidobacterium was planted into selective agar medium (BSM, Oxoid, USA). All plaques were incubated for 3-5 days at 37°C in anaerobic medium prepared in oxoid gas jars and anaerobic gaspak (Oxoid). The selective BSM medium was prepared by adding 50 mg mupirocin (Oxoid). Following incubation, the suspected bifidobacteria colonies detected by Gram reaction and colony morphology were cultured in 0.05% w/v L-cysteine-HCl (Merck) in modified Man, Rogosa and Sharpe broth medium (MRSc, Merck) anaerobically at 37°C for 24-48 h, and identified by anaerobic identification test kit (API 20A BioMerieux, France). Bacterial strains were stored in 10% glycerol at -80°C. For all tests, twice activated cultures were used.

Fructose-6-phosphate phosphoketolase enzyme activity and classic identification tests were used in the suspected bifidobacteria samples to identify the microorganisms to genus level (5). Test results were compared with the strains of *B. bifidum* (DSM 20456) and *B. breve* (DSM 20213) from the DSM culture collection.

### Determination of the probiotic properties of Bifidobacteria exopolysaccharide (EPS) production

Bacterial cultures were activated anaerobically at 37°C for 19 h, then boiled at 100°C for 10 min, cooled, and 85% trichloroacetic acid solution was added to the cultures to a maximum of 17% (v/v), centrifuged and cells and proteins were separated. Then, ethyl alcohol (96%, v/v) was added, centrifuged at 14,000 *g* for 20 min, at room temperature (23-25 °C) and EPS was precipitated. EPS (mg/L) was determined by the phenol-sulfuric acid method. A standard curve was formed with 5-100 mg/L glucose to identify EPS production quantity as per this standard.

### The effect of cholesterol on EPS production

To determine the effect of cholesterol on EPS production, the activated cultures were planted into media with and without 100 µg/mL cholesterol and incubated at 37°C for 19 h. Following incubation, the above mentioned EPS production method was applied.

### Determination of cholesterol elimination

For the cholesterol elimination study, the Gilliland et al. (16) modified method was used. The cholesterol solution previously prepared with 10 mg/mL ethyl alcohol and sterilized by filtration, was added to fresh MRSc liquid medium up to 100 µg/mL final concentrations. Then, this liquid was inoculated by 2% into the media for each strain, and incubated at 37°C for 19 h. Following incubation, cells were separated from the medium by a centrifuge [20 min at 10,000 *g*, at room temperature (23-25^o^C)]. The supernatant cholesterol amount was identified calorimetrically. A standard curve was formed with 10-150 µg/mL cholesterol to determine the cholesterol elimination amount. The formula A (%) = 100 - [(B/C) x 100] was used to determine the percent cholesterol elimination value of the strains [A: cholesterol elimination (%); B: cholesterol amount in the inoculated medium (µg/mL); C: cholesterol amount in the non-inoculated (control) medium (µg/mL)].

### Determination of bile salts deconjugation (taurocholic acid and glychocolic acid)

In the deconjugation study, 2 mg/mL sodium taurocholate (TCA) and sodium glycocholate (GCA) (Calbiochem, Germany) were added into MRSc mediums separately. Each strain was inoculated by 1% into the mediums, and incubated at 37°C for 18-20 h. No bacteria were added to the control groups, only 2 mg/mL TCA- or GCA-added media were used. Walker and Gilliland method (14), as modified by Irvin et al. (15) was used to identify TCA and GCA deconjugation amounts of the strains. A standard curve was formed with 100-1000 mg/mL colic acid (Calbiochem) to identify deconjugation amount, and colic acid concentration in the samples was identified as per this standard.

### Statistical analysis

Statistical analysis was performed using the SPSS 20.0 software (SPSS Inc., USA). All measurements were taken in triplicate. Data are reported as means±SD. The critical significance level for the statistical tests performed was set at 0.05. After assessing the normality distribution (Shapiro-Wilk test) and data homogeneity of variances, parametric *t*-test and ANOVA were used. In cases where these assumptions were not met, non-parametric Mann Whitney-U and Kruskal Wallis H-tests were used for comparison of differences between means. Pearson correlation coefficient was used to determine the association between tested parameters.

## Results

Thirty-five of the 94 fecal samples were positive for rotavirus antigen (37.23%). There was no rotavirus antigen in 59 of the samples. Seventy-four probable bifidobacteria samples were identified in the pink-violet mucoid colonial structures from the BSM selective medium added to the 35 GRV+ samples, 59 GRV- samples and 30 WG samples. F6FFK enzyme test was performed for pre-diagnosis of bifidobacteria in all isolates, and 59 isolates were found positive. Classical and anaerobic identification (API 20A) tests identified strains as follows, GRV+ samples: 8 *B. breve* and 5 *B. bifidum*; GRV- samples: 11 *B. breve,* 8 *B. bifidum*, and 1 *B. longum*; and in the WG samples: 14 *B. breve*, and 12 *B. bifidum*.

**Figure 1:**
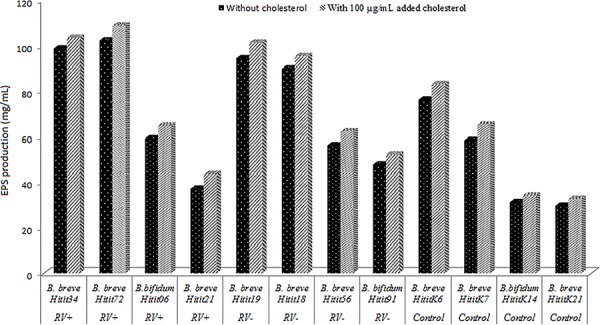
Exopolysaccharide (EPS) produced by bifidobacteria strains isolated from rotavirus-positive with gastroenteritis (RV+), rotavirus-negative with gastroenteritis (RV-) and without gastroenteritis (Control) samples, with and without added cholesterol.

**Figure 2:**
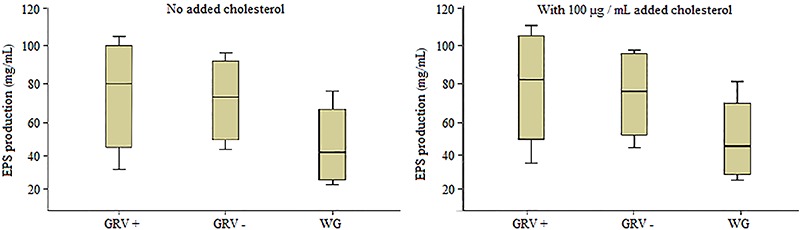
Effect of added cholesterol on exopolysaccharide (EPS) produced by bifidobacteria isolated from rotavirus-positive with gastroenteritis (GRV+), rotavirus-negative with gastroenteritis (GRV-) and without gastroenteritis (WG) groups.

All strains produced different quantities of EPS. EPS production by the bifidobacteria isolated from GRV+, GRV-, and WG ranged between 29.56-102.21 mg/mL. As presented in Table 1, GRV+ bifidobacteria strains generally produced higher EPS compared to GRV- and WG groups. A significant difference for EPS production was observed among groups (P<0.05). Also, cholesterol had a positive impact on EPS production of all bifidobacteria strains in the media. EPS production was 29.56-102.21 mg/mL if cholesterol was not added into the media (0 µg/mL), and increased to 32.65-108.56 mg/mL, when cholesterol was added into the media (100 µg/mL; Figures 1 and 2). A strong positive correlation was found between EPS production and cholesterol (r=0.984, P<0.001). From the data, the highest and lowest EPS-producing strains were selected from each group, and cholesterol elimination and bile salts (TCA and GCA) deconjugation were studied in a total of 12 strains.

**Table:**
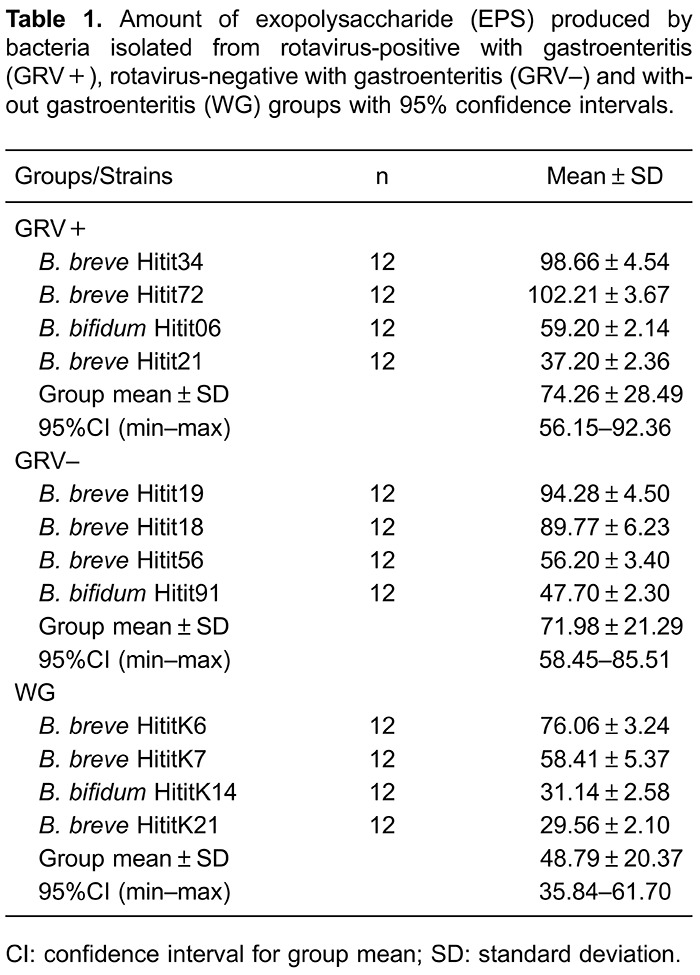

The 12 bifidobacteria strains that produced EPS had different cholesterol elimination capacities from the medium. The cholesterol elimination rate ranged between 8.36-39.22% for the 19-h incubation period. Table 2 shows that the cholesterol elimination rate of GRV+ bifidobacteria strain was higher compared to GRV- and WG groups. Significant differences were observed among groups for cholesterol elimination (P<0.05). When TCA (7.5-31.5%) and GCA (9.4-33.5%) deconjugation rates were compared, the GCA deconjugation rate was higher (Table 3). A significant difference was observed among groups for both TCA and GCA deconjugation (P<0.05).

**Table:**
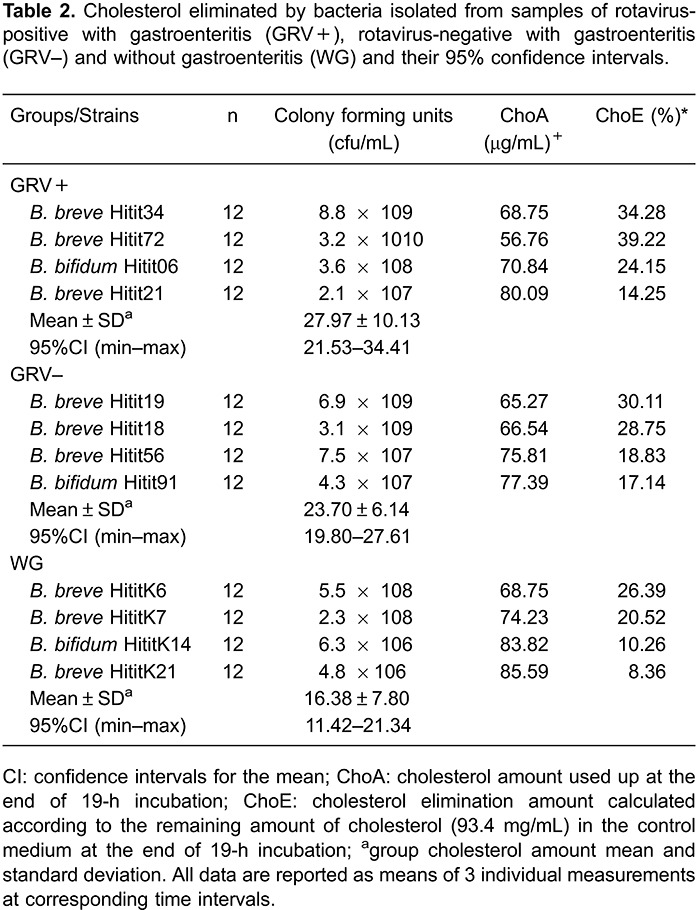

**Table:**
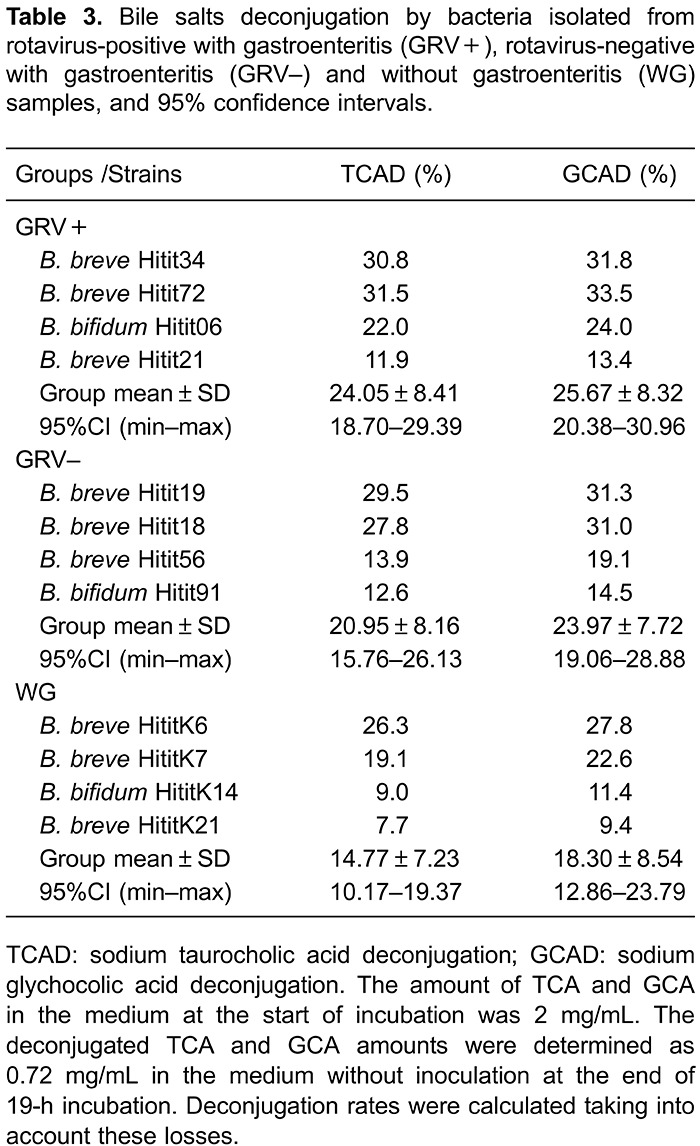

Using the Kruskal-Wallis H test, a significant difference was observed among groups in all tested parameters (P<0.05). A significant strong positive correlation was found among all parameters (EPS production, cholesterol elimination, TCA and GCA deconjugation; Table 4).

**Table:**
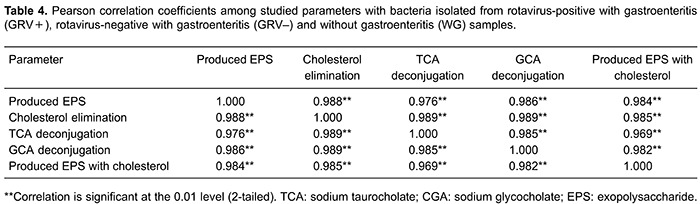

## Discussion

Bifidobacteria colonizes the intestinal surface during the first days after birth, and continues to be a member of intestinal flora in humans and animals throughout life (17). These bacteria are predominant especially in the intestinal flora of lactating babies (18), and are considered to be beneficial and important for a balanced normal intestinal flora. However, viruses and bacteria causing gastroenteritis sometimes disturb the balance of the intestinal microflora. In recent years, rotavirus gastroenteritis, especially common in developing countries, is considered to be associated with significant morbidity and mortality in children below 5 years of age (19–21). Studies have shown that increased bifidobacteria in the intestines prevent proliferation of exogenous pathogens (22,23). Therefore, the primary aim of our study was to determine bifidobacteria flora in children with rotavirus infection, and to determine the important probiotic properties of these bacteria. The data of this extensive study showed that a total of 33 bifidobacteria were isolated from 94 children with gastroenteritis symptoms. However, 26 bifidobacteria were identified from healthy children below 5 years of age, which were used to compare probiotic properties of the isolated bifidobacteria. The bile salts deconjugation capacity and cholesterol elimination were compared in the strains with highest and lowest EPS production of the isolated and identified bifidobacteria from the 3 groups (GRV+, GRV−, and WG).

Several investigators have reported that lactic acid bacteria produce EPS, and there are a few studies on EPS production of bifidobacteria (23). Most of these studies focused on the structure and characterization of EPS. In general, these studies did not emphasize probiotic properties of EPS production capacity, which was an important aim of our study. Some studies showed that bifidobacteria (especially *B. breve* strains) have high EPS production capacity (24). Moreover, EPS is beneficial for the protection of bacteria against gastric acid and bile and thus, bacteria can reach the intestines safely (25,26). Therefore, lactic acid bacteria are very important in milk and dairy technology due to their viscosity, rigidity, stability and stabilizer properties (27,28). Commercial probiotic products with EPS production capacity help bacteria reach the intestine. In our study, all isolated bifidobacteria had different rates of EPS production capacity. However, EPS production was higher in children with rotavirus infection compared to other groups.

Bile salt hydrolase (BSH) is the enzyme responsible for bile salt deconjugation during enterohepatic circulation in healthy people. There are six major bile acids including TCA released from taurine, glycine and free bile salts, taurodeoxycholic acid, taurochenodeoxycholic acid, GCA, glycodeoxycholic acid and glycochenodeoxycholic acid (28,29). Deconjugated bile salts are not soluble in water, and are eliminated predominantly via feces (30). Most of the bacteria use amino acids for energy. BSH activity is an important factor for the intestinal colonization of enteric bacteria including lactobacilli and bifidobacteria (31,32). There are extensive studies on this issue. One of them is the study by Tanaka et al. (11), in which more than 300 lactic acid bacteria had BSH activity and distribution. Our study used sodium taurocholate and sodium glycocholate, and yielded different ratios of deconjugation. However, the deconjugation rates were in line with EPS production. In recent years, BSH enzyme was shown to play an important role on cholesterol metabolism due to its effect on serum cholesterol levels (32,33). Further study of bile salt deconjugation and cholesterol elimination from media when selected probiotic microorganisms with cholesterol lowering effects is recommended (33,34).

Most of the studies in the literature suggest specific probiotics to be used as additive treatment in infectious diarrhea, but few of them report on the treatment efficacy of probiotics. In a study performed by Isolauri et al. (35), it was reported that *Lactobacillus casei* sp. strain GG in the form of milk or freeze-dried powder is effective in short-term treatment for acute diarrhea (82% rotavirus) in 4-45 months-old children. In a similar study on *Lactobacillus* GG strain, viral and bacterial diarrhea were studied, however, that strain shortened the duration of the rotavirus diarrhea only; no efficacy was identified on bacterial diarrhea (36).

Protection against rotavirus infection includes improvement of the supplied water quality, hygiene, food sanitation and vaccination. Rotavirus vaccine decreases the hospitalization rate and financial burden of the disease, especially when costs of outpatient clinics are compared (37,38). The World Health Organization gives priority to rotavirus vaccine; however, it has not been included in the national vaccine program in most of the underdeveloped and developing countries. Therefore, alternative ways including effective nutrition becomes crucial as a treatment. Probiotics are viable microorganisms in the gastrointestinal microbiota of the host. Currently, the role of the gut flora in host metabolism and immune systems of children emphasizes the importance of developing probiotic technology (39,40). Our study suggests that superior probiotic microorganisms isolated from humans may be used as a supplement in milk and dairy products to decrease mortality and morbidity associated with rotavirus diarrhea. An important finding of our study is that the bifidobacteria survived the rotavirus infection, and their probiotic properties were superior compared to the bifidobacteria from healthy individuals and to those who were not infected with rotavirus. The strongest bacteria in the intestinal microbiota survived the infection even though important components had been lost. The resistance and superior probiotic properties of the survivors make them more valuable. Even though there are several commercial probiotic products on the market, the superior bifidobacteria that we studied may play an important role in decreasing the contamination and minimizing the effects of rotavirus infection. In conclusion, given the increased numbers of rotavirus infection cases and the poor availability of treatment methods in Turkey and other countries, it is very important to add superior and resistant bifidobacteria to the diets of the children infected with rotavirus to improve the intestinal and vital functions.
